# V5 region in the HIV-1 envelope glycoprotein determines viral sensitivity to the broadly neutralizing monoclonal antibody VRC01

**DOI:** 10.1186/1742-4690-9-S2-P46

**Published:** 2012-09-13

**Authors:** D Guo, X Shi, K Arledge, D Song, L Jiang, L Fu, S Zhang, X Wang, L Zhang

**Affiliations:** 1 Institute of Pathogen Biology,Chinese Academy of Medical Sciences , Beijing, China; 2 Comprehensive HIV/AIDS Research Center, Tsinghua University , Beijing, China; 3 Comprehensive HIV/AIDS Research Center,Tsinghua University , Beijing, China; 4 China Agricultural University, Beijing, China; 5 School of Medicine,Tsinghua University , Beijing, China

## Background

VRC01, a broadly neutralizing monoclonal antibody (bnmAbs), is capable of neutralizing a diverse array of HIV-1 isolates through recognition of the loop D and the V5 regions within the CD4 binding site on envelope glycoprotein gp120. Nonetheless, resistant strains have been identified. Here, we examined two closely related envelope clones derived at a single time point from a CRF08_BC infected patient which displayed an over 20-fold difference in VRC01 neutralization sensitivity.

## Methods

A total of 15 chimeric envelope clones were generated by interchanging the loop D and/or V5 regions between the original envelopes or by single alanine substitutions within each region. The resultant effects on VRC01 neutralization sensitivity were subsequently studied in the context of pseudotyped viruses.

## Results

Our results showed that interchanging the V5 region between the two clones completely swapped their neutralization sensitivity profiles, while exchanging the loop D region alone had minimal impact. Mutagenesis analysis revealed that the potential N-linked glycosylation site (PNGS) at position 460 in the V5 region contributed to over 90% of observed resistance, while other amino acid changes made no discernible differences. Furthermore, changes in resistance were found to positively correlate with VRC01 binding activity to the corresponding envelope glycoprotein. None of the substitutions, however, significantly altered binding and neutralization sensitivity to bnmAb b12 or soluble CD4. Of note, a mutation that removed the PNGS at position 463 in the V5 region increased resistance to ibalizumab, a non-immunosuppressive monoclonal antibody that binds CD4 and has been shown to inhibit entry of diverse HIV-1 isolates.

## Conclusion

In summary, our data indicates that amino acid residues in the V5 region play a critical role in determining viral sensitivity to VRC01. Increased length, glycosylation and long side-chain of amino acids in the V5 region may collectively create steric hindrance that lowers binding affinity, thereby increasing resistance to VRC01 neutralization.

